# Influence of Proximal Box Design on Primary Stability and Strength of Indirect Partial Bonded Restorations (IPBR) in Posterior Teeth

**DOI:** 10.3390/medicina62101872

**Published:** 2026-09-27

**Authors:** Mariangela Cernera, Ida Papallo, Vito Gallicchio, Vincenzo Spinelli, Francesco Traino, Rosario Schiano Lo Moriello, Gaetano Paolone, Salvatore Scolavino

**Affiliations:** 1 Department of Neurosciences, Reproductive and Odontostomatological Sciences, University of Naples Federico II, 80131 Naples, Italy; fr.traino@hotmail.com; 2 Department of Industrial Engineering, University of Naples Federico II, 80125 Naples, Italy; ida.papallo@unina.it (I.P.); rosario.schianolomoriello@unina.it (R.S.L.M.); 3 Centro Servizi Metrologici e Tecnologici Avanzati (CeSMA), University of Naples Federico II, 80146 Naples, Italy; vincenzo.spinelli@unina.it; 4 Restorative Dentistry, Department of Health Science, Unicamillus—Saint Camillus International Medical University, 00131 Rome, Italy; gaetano.paolone@unicamillus.org

**Keywords:** mechanical measurements, finite element analysis, bond strength, proximal box design, partial bonded restorations

## Abstract

*Background and Objectives*: To evaluate the influence of proximal box design on primary stability and adhesive bond strength of indirect partial bonded restorations (IPBR) in posterior teeth. *Materials and Methods*: Thirty extracted human third upper molars were randomly assigned to three groups (*n* = 10 each): Group A, butt joint overlay-type restorations with proximal box (height-to-depth ratio 1:2–1:3); Group B, proximal box with height-to-depth ratio 1:1; and Group C, flat bevel preparation as control. Specimens underwent standardized preparation: immediate dentin sealing, restoration with overlays, and cementation with Panavia V5 adhesive resin cement. Mechanical measurements were performed to assess the tensile bond strength 48 h post-cementation using a universal testing machine. Finite element analysis (FEA) was also carried out to evaluate stress distribution at the adhesive interface. Data were analyzed by ANOVA with the Bonferroni post hoc tests. *Results*: Group A exhibited significantly higher bond strength values (403.7 ± 37.5 N) compared with Group B (340.4 ± 32.7 N; *p* < 0.01) and Group C (324.8 ± 33.3 N; *p* < 0.01). No significant difference was observed between Groups B and C. FEA corroborated the experimental findings, showing more homogeneous stress distribution for Group A and higher stress concentrations in Groups B and C. *Conclusions*: Proximal box design significantly affects the primary stability and bond strength of IPBR in vitro. A deeper proximal box with favourable geometric features enhances adhesive performance and stress distribution. Within the limitations of this study, these findings suggest potential clinical benefits, although further in vivo studies under dynamic conditions are needed to confirm long-term restoration performance.

## 1. Introduction

In contemporary dental practice, resin composites are widely used for a variety of restorative procedures, supporting a minimally invasive approach aimed at preserving tooth structure and optimizing patient care. Structurally compromised vital teeth can be restored using either direct or indirect restorations made from different materials. The choice of the most appropriate treatment depends primarily on structural factors and the need to preserve tooth vitality.

Loss of dental structure may result from several factors, including caries, dental fractures, large or defective amalgam restorations, and endodontic treatment. These conditions may necessitate cusp reduction and coverage, particularly when the residual cusp or wall thickness is less than 1.5 mm in vital teeth and 2.5 mm in non-vital teeth [1]. Endodontically treated teeth, in particular, show increased risk of vertical fracture due to weakened structure [2,3,4,5,6]. Disrupting the continuity of the tooth structure reduces rigidity, with cusp flexibility increasing as more tooth material is removed, especially following endodontic treatment [7].

To reduce the risk of tooth fracture, cusp reduction and occlusal covering is recommended using partial indirect restorations known as overlays. Indirect partial bonded restorations (IPBR) represent a minimally invasive approach to restore heavily compromised posterior teeth, as overlay preparations enable the preservation of over 50% of healthy tooth structure compared to full crowns [8,9].

Different overlay preparation designs have been proposed in the literature, with particular emphasis on occlusal and proximal preparation geometry. Although a flat preparation results in a lower C-factor, thereby reducing polymerization stress within the luting composite compared with cavity preparations featuring a more complex configuration [10], a flat preparation has some limitations and shortcomings. First, a flat preparation does not provide an auto-centering effect, making reliable and reproducible seating of the restoration more challenging. In addition, during the luting procedure, excess luting composite may cover the finishing line, making it difficult to verify the complete and correct seating of the overlay. Finally, the absence of a proximal box may reduce the primary stability of the restoration.

Overlay cavity preparations can be executed with or without proximal boxes, highlighting a critical aspect of the design process [9,11,12,13]. However, to the authors’ knowledge, no previous study has investigated the influence of the depth and the thickness of the proximal box on the primary stability of the restoration.

Various materials, including ceramics, composites, and zirconia, have been proposed for IPBR, each exhibiting unique properties. While scientific evidence supports the adhesion of indirect partial zirconia restorations, controlled clinical trials are essential to determine the strength and durability of the achieved bond, especially in non-retentive preparations [14,15,16].

Hence, the objective of this in vitro study is to assess how the presence and design of the proximal box, as well as its thickness and depth, influence the primary stability and bond strength of IPBRs, thereby providing significant insights into a clinically relevant aspect of contemporary restorative dentistry through mechanical measurements and finite element analysis (FEA).

The null hypothesis was that proximal box designs do not influence the primary stability of the restoration or the stability of the adhesive cementation.

## 2. Materials and Methods

The collection and use of human teeth for this in vitro study were approved by the Ethics Committee of Campania 3 (Protocol No. 299/2024, 5 March 2025), and written informed consent was obtained from all subjects prior to participation.

The initial sample size estimation was guided by the previous literature in the field. Specifically, we referenced the study by [Kasem, Ammar T et al., 2024] [17], which reported a large effect size of Cohen’s d = 2.05 and a sample size of 10 per group.

Thirty intact, caries-free, non-restored human third upper molars from individuals aged 16–40 years old (ISO/DTS 11405 Dentistry), extracted for therapeutic reasons, were selected. To ensure specimen standardization, only teeth with fully developed roots and similar crown dimensions—specifically, a mesiodistal diameter of 8.5 ± 0.5 mm and a buccolingual diameter of 10.0 ± 0.5 mm—were included in the study. After extraction, all teeth were debrided from residual plaque and calculus and, according to ISO/DTS 11405 [18], were stored in phosphate-buffered salt solution of neutral pH at 4–7° throughout the entire experimental process for a maximum of six months to avoid tooth dehydration.

### 2.1. Tooth Preparation

Teeth were randomly assigned to one of the three groups (A, B, C), with 10 teeth in each group. All preparations were performed by the same experienced operator (S.S.). The preparation process involved the use of magnifying glasses (3×), a periodontal probe (CP-15, Hu-Friedy Italy SRL, Milan, Italy), and rounded taper diamond burs mounted on a red ring contra-angle handpiece with water cooling. The following burs were used sequentially:

ISO 806 314 305 524 014 (Intensiv–Swiss Dental Products, Montagnola, Switzerland) for 2 mm of occlusal reduction.

ISO 806 314 609 514 010 (Intensiv–Swiss Dental Products, Montagnola, Switzerland) to create the proximal boxes.

ISO 806 314 370 514 008 (Intensiv–Swiss Dental Products, Montagnola, Switzerland) to create a circumferential chamfer (Group C) or buccal and lingual chamfer connecting the boxes from the mesial to the distal side box to box (Group A and B).

FG Silicon bur Brownie Mini-Points 0413 (Shofu—Kyoto, Japan) to finish the preparation surface.

To maintain cutting capability and prevent wear of the diamond grit, a new bur was used for each group. The initial preparation step involved a 2 mm occlusal reduction, following the inclination of the cusp and the main sulcus. This reduction ensured the appropriate thickness for the prosthetic overlay. The cavity preparations were completed as follows (Figure 1):

Group A (Test 1): butt joint overlay-type restorations with buccal and lingual chamfers and an interproximal box with a 1:2 or 1:3 height-to-depth ratio;

Group B (Test 2): butt joint overlay-type restorations with buccal and lingual chamfers and an interproximal box with a 1:1 height-to-depth ratio;

Group C (Control): flat bevel used as the standard preparation.

### 2.2. Adhesive Protocol and Provisionalization

At the end of the preparation, the preparation surface was treated with a dedicated air polisher (Air- Flow SII, EMS, Nyon, Switzerland) using 20 microns of glycine powder in order to remove FG silicon bur residual parts.

Thereafter, according to IDS [19], ortho-phosphoric acid at 37% (K Etchant—Kuraray Noritake) was applied on the enamel of the dried cavity preparation for 20 s. After rinsing the acid for 20 s, the adhesive system Clearfil Universal Bond Quick (‘C-UBq’; Kuraray Noritake, Tokyo, Japan) was applied on the dried cavity. The application involved twice active rubbing for a total of 40 s. Subsequently, air drying for 10 s and light curing for 40 s at 1100 mW/cm^2^ using Valo X light cure device (Ultradent Products, Inc., South Jordan, UT, USA) with intensity monitoring were performed.

To prevent the hydrolytic effects induced by water exposure, all treated surfaces were coated with Clearfil Majestic Es Flow High Viscosity (Kuraray Noritake, Tokyo, Japan), applied directly from the syringe and spread in an extremely thin layer with the sharpened tip of a composite instrument. Polymerization of the flowable composite was followed by the application of an air-blocking barrier (glycerin jelly) and 20 s of additional light exposure with the same light-curing unit to polymerize the oxygen-inhibition layer. After the scanning step, the bonded surfaces were then isolated with petroleum gel. Teeth were restored with a light-curing temporary restorative material (Telio CS Onlay; Ivoclar Vivadent-Liechtenstein, Ivoclar Vivadent, Amherst, NY, USA), left in place for 2 weeks before the final restoration, and stored in saline solution.

### 2.3. Zirconia Overlays

All indirect restorations were fabricated in a certified dental laboratory following the manufacturer’s instructions (Kuraray Noritake, Japan). Monolithic zirconia overlays were produced using a multilayered zirconia material (Katana STML A2, height 14 mm; Kuraray Noritake, Japan), selected for its enhanced translucency and esthetic properties suitable for partial indirect restorations. The restorations were designed using dental CAD (Computer-Aided Design) software (DentalCAD 3.3 Chemnitz, Exocad DentalCAD GmbH, Darmstadt, Germany) based on the STL files obtained from intraoral scanning 3Shape Trios (3Shape, Copenhagen, Denmark). Subsequently, the overlays were milled using a 5-axis milling unit (Programill PM7, Ivoclar Vivadent, Liechtenstein) under dry conditions in order to avoid contamination and potential interference with the sintering process. After milling, the restorations underwent sintering in a high-temperature furnace (Dekema 674i, Dekema Dental-Keramiköfen GmbH, Freilassing, Germany), equipped with advanced thermocouple control to ensure precise temperature regulation.

The sintering cycle was performed according to the manufacturer’s recommended protocol for Katana zirconia, ensuring complete densification and optimal mechanical properties. Following sintering, the restorations were finished and polished using zirconia-specific diamond burs.

### 2.4. Surface Treatment and Cementation

Provisional restoration was removed, and the entire preparation surface was cleaned by airborne-particle abrasion (AquaCare Twin, Velopex, London, UK). Thereafter, the enamel was etched with 37% phosphoric acid (K Etchant-Kuraray Noritake-Japan) for 20 s and rinsed with water for 20 s. Panavia V5 Tooth Primer (Kuraray Noritake-Japan) was applied on the dried cavity surfaces, and the excess was removed with gentle air blow.

The overlays’ intaglio surfaces were air-abraded with 50 μm (micron) alumina particles at 2 bar pressure, at a distance of 10/20 mm, at an angle of 45–75°, for 10 s. After vaporization, the overlays were dried, and finally a 10-MDP silane coupling agent (Ceramic Primer Kuraray Noritake-Japan) was applied [14,20,21].

The 10-MDP adhesive resin-based cement Panavia V5 (Kuraray Noritake-Japan) was used to lute the overlays on teeth. After removal of excess luting, the specimens were light-cured for 40 s followed by the application of an air-blocking barrier (glycerin jelly) and 20 s of additional light exposure.

### 2.5. Reverse Engineering–3D Scanning

The sound teeth were scanned by a three-dimensional dental intraoral scanner Trios3 3Shape Wired (3Shape, Copenhagen, Denmark) before and after preparation with and without IDS and after luting of the overlays. All the STL files were imported into laboratory management software (Exocad DentalCAD GmbH).

### 2.6. Mechanical Measurements

The specimens were tested following a procedure already reported in the literature [22]. Specifically, ten specimens for each group were subjected to tensile bond strength testing 48 h after cementation. In brief, each specimen was mounted in acrylic resin to ensure stability during testing. The mounted specimens were successively positioned in a universal testing machine (INSTRON). The bond strength measurements were performed employing such an experimental setup. The acrylic resin blocks were properly held in place by securing the specimen with two clamps and tested at a crosshead speed of 0.5 mm/min until failure. The peak load (i.e., the maximum force, F_max_) was recorded and reported as mean value ± standard deviation.

### 2.7. Finite Element Analysis (FEA)

3D geometrical models of the restored teeth were first built according to each preparation design and then imported into an FE pre-processor, which allowed the management and the generation of complex models. The materials/components of each model were properly defined in terms of Young’s modulus and Poisson’s ratio.

Based on previous methodological approaches [23,24,25], the 3D mesh was created, and each tooth model was divided into 3D solid CTETRA elements with four grid points.

An appropriate mesh size and refinement techniques were employed. As for contact elements, “freeze” type elements were used as the contact condition between the several parts.

Thus, 3D models were constructed (Model A, Model B and Model C) according to the different components (Figure 2).

Finite element analysis (FEA) was performed on the three models (Model A, Model B and Model C) to simulate the experimental mechanical analysis.

The maximum principal stress and shear stress distributions were analyzed at the adhesive interface layer.

### 2.8. Statistical Analysis

After checking for normal and equal distributions (Shapiro–Wilk and Brown–Forsythe tests (*p* > 0.05), respectively), a one-way ANOVA with the Bonferroni post hoc test was applied. Statistical significance was considered for *p*-values < 0.05.

An a priori power calculation was performed to justify the sample size. Additionally, to evaluate whether our final sample size provided sufficient statistical sensitivity, a post hoc power analysis was performed. This post hoc analysis is presented merely as supportive evidence rather than a substitute for the primary a priori power calculation. Based on the final sample size (*n* = 30), an alpha level of α = 0.05, and the observed large effect size (d = 2.05), the achieved statistical power (1 − β) for the one-way ANOVA with the Bonferroni post hoc test was calculated to be 0.95 (95%).

All tests were performed using SigmaPlot v. 14.0 (Systat Software GmbH, Düsseldorf, Germany).

## 3. Results

### 3.1. Mechanical Measurements

Table 1 reports the results from mechanical measurements in terms of tensile bond strength (F_max_).

However, the *p*-value corresponding to the F-statistic of one-way ANOVA was lower than 0.05, thus suggesting that one or more treatments were significantly different.

Accordingly, the Bonferroni post hoc test was carried out to identify which of the pairs of treatments were significantly different from each other.

In particular, Group A exhibited significantly higher bond strength (403.7 ± 37.5 N) compared with Groups B (340.4 ± 32.7 N) and C (324.8 ± 33.3 N) (*p* < 0.01). No significant difference was observed between Groups B and C. Furthermore, with regard to Group A, it is also worth stating that in terms of F_max_ no statistically significant difference was found between interproximal boxes with a 1:2 and 1:3 height-to-depth ratio.

### 3.2. Finite Element Analysis (FEA) Outcome

The FEA results allowed us to analyze the maximum principal stress and shear stress distributions at the interface layer for both the “lower” and “upper” surfaces (Figure 3, Figure 4 and Figure 5).

The colour scale was suitably chosen to allow comparisons among the models.

Figure 3, Figure 4 and Figure 5 clearly highlight the differences in terms of stress distributions at the adhesive interface layer (for both upper and lower surfaces) among the models A, B and C.

Basically, as shown in the results from FEA, Model A (i.e., Group A in the experimental analysis) showed the most homogeneous stress distribution at the adhesive interface, with reduced stress concentrations.

On the other hand, Model B (i.e., Group B in the experimental analysis) displayed localized stress concentrations, particularly at marginal zones of the proximal boxes, whilst Model C (i.e., Group C in the experimental analysis) revealed the least favourable pattern, with pronounced stress concentrations along the margins.

Such findings corroborated the experimental evidence, highlighting the mechanical and structural advantages of deeper proximal box designs.

## 4. Discussion

The present investigation demonstrated that proximal box design significantly influences the adhesive bond strength and stress distribution of indirect partial bonded restorations (IPBR); therefore, the null hypothesis is rejected. Preparations incorporating a proximal box with a height-to-depth ratio of 1:2–1:3 achieved higher tensile bond strength than shallower proximal boxes or flat designs, and finite element analysis (FEA) corroborated these findings, showing more homogeneous stress distributions and reduced stress concentrations at the adhesive interface. These results reinforced the central role of cavity geometry in primary stability and in the reliability of adhesive cementation.

Our data align with consolidated evidence that both the amount and configuration of tooth structure removal affect biomechanical behaviour and restoration longevity. Classic studies emphasized how the preparation geometry and C-factor drive polymerisation stress and interfacial stress concentration [9,10], while morphology-driven preparation concepts further argued that well-defined proximal features can improve seating, centring, and primary stability without sacrificing tissue preservation [12,13]. More recent computational studies investigating proximal and box-like cavity configurations have shown that both the magnitude and distribution of stresses are strongly influenced by cavity geometry. Appropriate preparation designs can reduce interfacial tensile and shear stresses, regardless of the restorative material used [25,26].

A clinically relevant corollary emerges from the literature on proximal box elevation (PBE)/deep margin elevation (DME). In vitro evidence shows that elevating/substituting the proximal box floor does not impair, and can even improve, the bond strength of composite inlays to the proximal floor—though the magnitude of improvement may depend on the cement used [27]. Moreover, in endodontically treated premolars restored with ceramic endocrowns, PBE increased fracture resistance without increasing microleakage, supporting the notion that a more favourable geometry near the proximal margin enhances mechanical performance without compromising sealing [28].

These clinical findings are consistent with the present in vitro results, showing that a geometrically adequate proximal box (Group A) provides a measurable mechanical advantage. In contrast, a shallow box (1:1; Group B) performs similarly to a flat preparation (Group C), suggesting a threshold effect in which insufficient box depth does not substantially modify interfacial mechanics [27].

From a mechanical point of view, a deeper proximal box likely contributes to better geometric interlocking, a greater adhesive area with a more favourable orientation of bonded interfaces, and improved seating auto-centering during luting, all of which diminish micro-movement under load and attenuate local stress peaks. These mechanisms are consistent with findings from the broader FEA literature, in which parametric analyses of inlay and onlay configurations have demonstrated that specific cavity features, including isthmus width and proximal box depth and geometry, significantly influence peak principal and shear stresses within the enamel, dentin, and the adhesive interface. Appropriately dimensioned preparation designs may reduce unfavourable stress concentrations and promote a more homogeneous distribution of functional loads [27]. In addition, broader FEA-based and experimental work on indirect ceramic/composite partial restorations has shown that concave or optimized cavity forms can constrain stress in dentin and mitigate catastrophic failure modes—findings that conceptually align with our observation that Group A reduces interfacial stress concentration compared with Groups B and C [1]. These results suggest incorporating adequately deep and proportioned proximal boxes in IPBR when proximal defects or subgingival margins are present, as part of a minimally invasive strategy that seeks to preserve tissue yet optimize biomechanics [1,8,15]. This recommendation is particularly relevant for endodontically treated or structurally compromised teeth, in which reduced structural integrity may increase susceptibility to vertical fracture [2,3,4,5,6]. DME-oriented protocols can complement this approach by relocating margins to a more manageable level without undermining adhesion, as originally advocated in the DME concept and reiterated by subsequent analyses [29].

These limitations should be considered when interpreting the clinical relevance of the present findings. First, the study was conducted in vitro under static loading conditions 48 h after bonding [30], whereas the intraoral environment exposes restorations to cyclic mechanical loading, thermal and humidity fluctuations, and hydrolytic degradation of the adhesive interface. These factors may adversely affect the long-term stability of the adhesive joint. Although immediate dentin sealing (IDS) protocols can improve adhesive performance, they cannot completely prevent the degradation processes that occur over time [19,31]. Furthermore, FEA simplified the materials as linear elastic and isotropic under static loads; future modelling should incorporate viscoelastic features, moisture-dependent properties, and fatigue. Finally, the sample size, although sufficient to detect the reported effects, limits subgroup analyses across tooth types, adhesive systems, and restorative materials.

Within these limitations, the agreement between our experimental and computational findings, together with supporting evidence from the PBE/DME and FEA literature, justifies rejection of the null hypothesis. As a result, proximal boxes should be designed with adequate depth (approximately a 1:2–1:3 height-to-depth ratio) to improve primary stability, reduce interfacial stresses, and enhance the adhesive performance of posterior IPBRs. Prospective studies including adhesive failure mode, thermomechanical ageing, fatigue, and randomized clinical trials should verify whether the in vitro gains observed here translate into superior clinical longevity.

## 5. Conclusions

Within the limitations of the current in vitro study, the following conclusions can be drawn:

1. Proximal box design significantly affects the primary stability and adhesive performance of indirect partial bonded restorations (IPBR). Preparations incorporating a proximal box with a 1:2–1:3 height-to-depth ratio (Group A) exhibited superior bond strength and more favourable stress distribution compared with shallower boxes or flat designs.
2. Finite element analysis (FEA) corroborated the experimental findings, showing that deeper proximal boxes provide a more homogeneous stress distribution at the adhesive interface while also reducing stress concentrations.

## Figures and Tables

**Figure 1:**
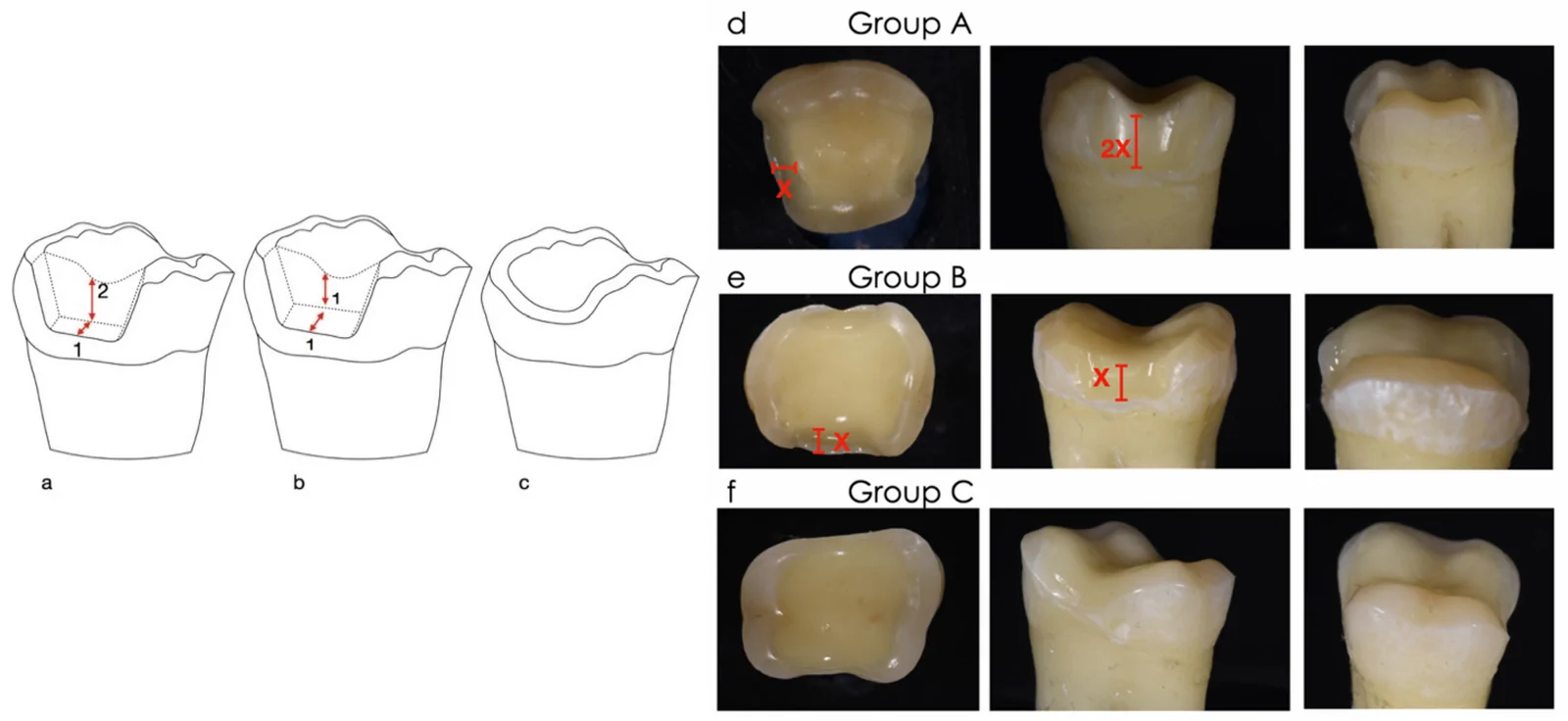
Schematic representation of Group A (**a**), Group B (**b**), and Group C (**c**). Final tooth preparations corresponding to Group A (**d**), Group B (**e**), and Group C (**f**).

**Figure 2:**
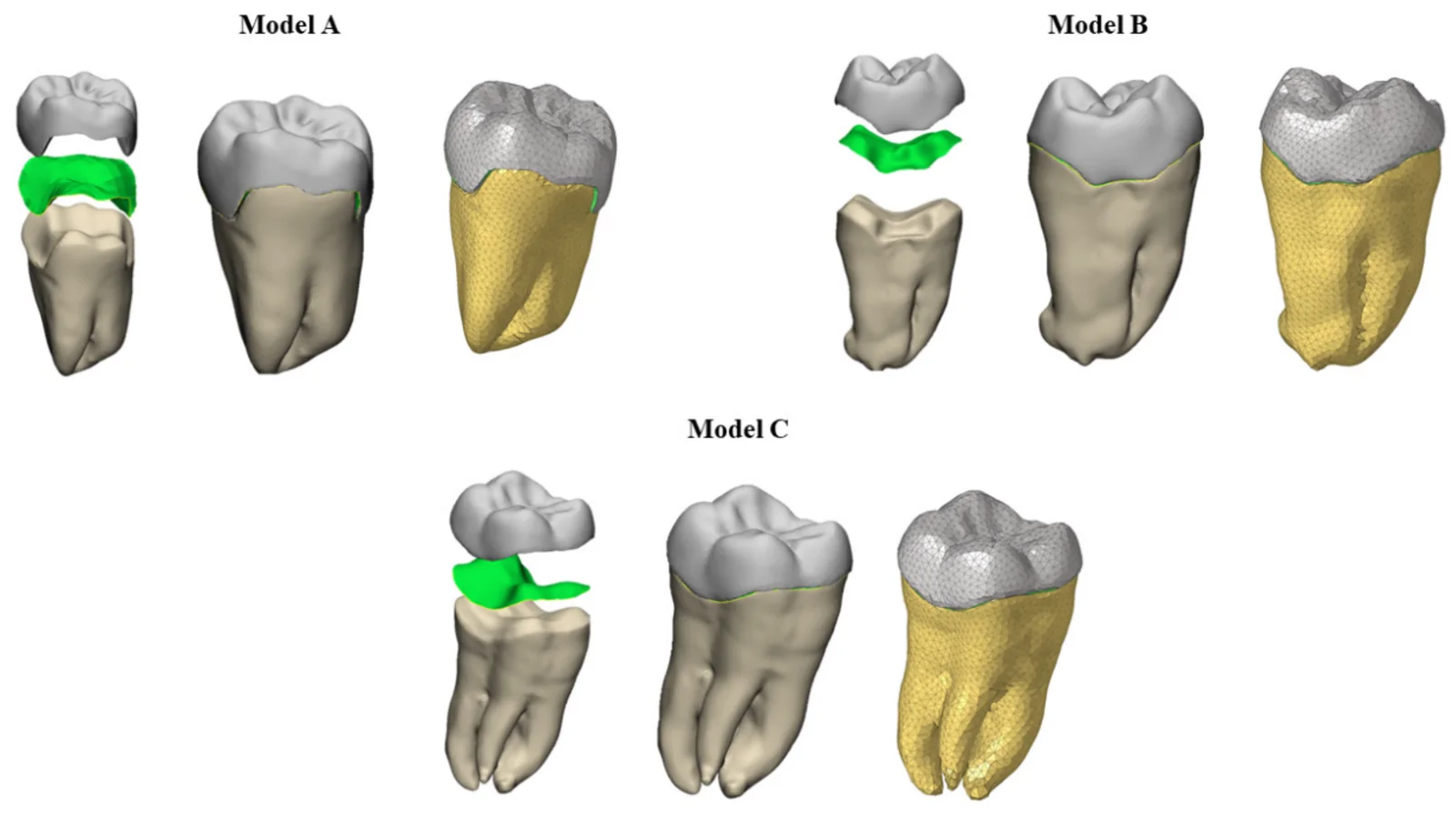
Different components and 3D geometrical models for finite element analysis.

**Figure 3:**
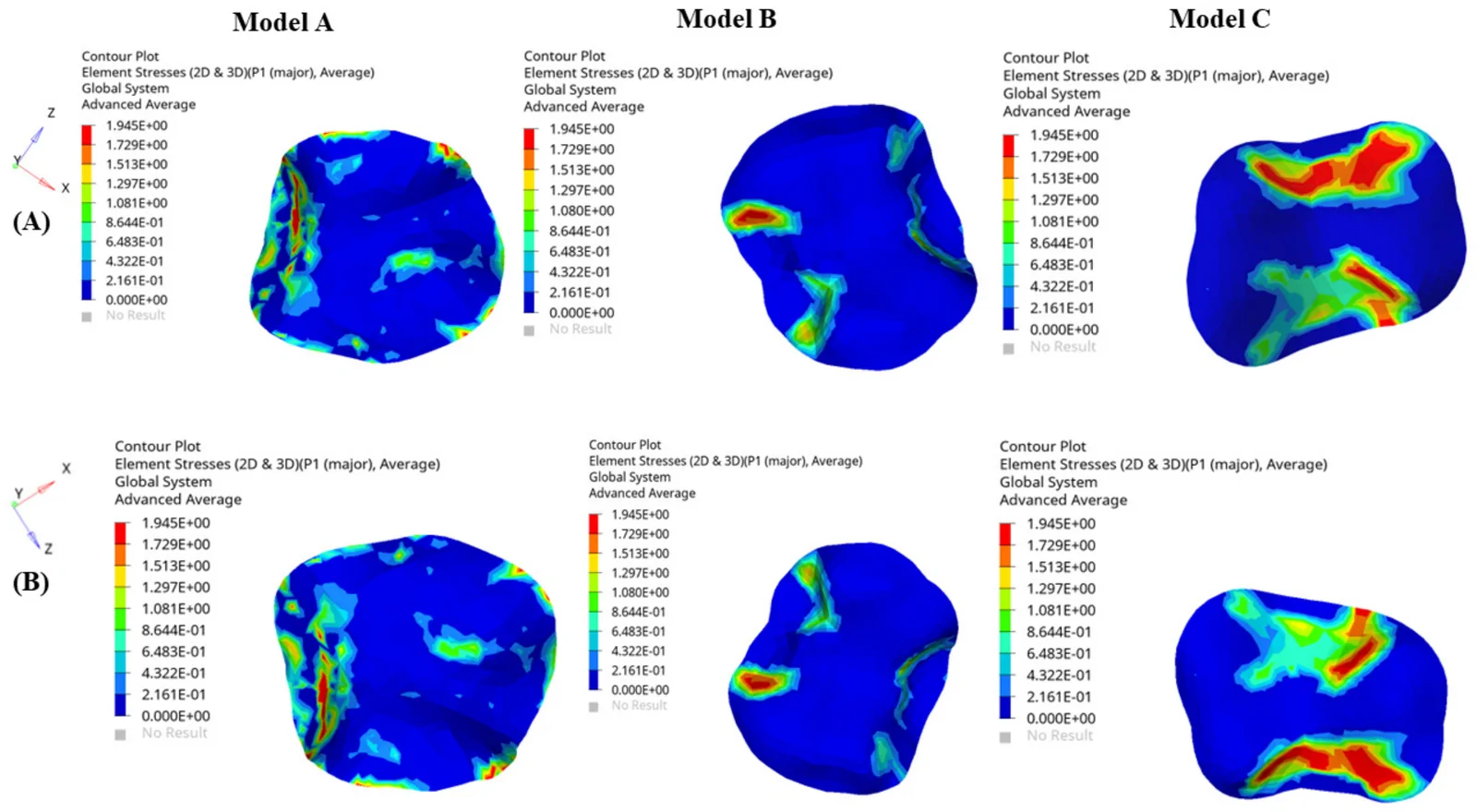
Results from FEA on models A, B, and C: maximum principal stress distribution (MPa) at the adhesive interface layer: (**A**) lower surface; (**B**) upper surface. The colour scale was properly selected to allow comparisons among the models.

**Figure 4:**
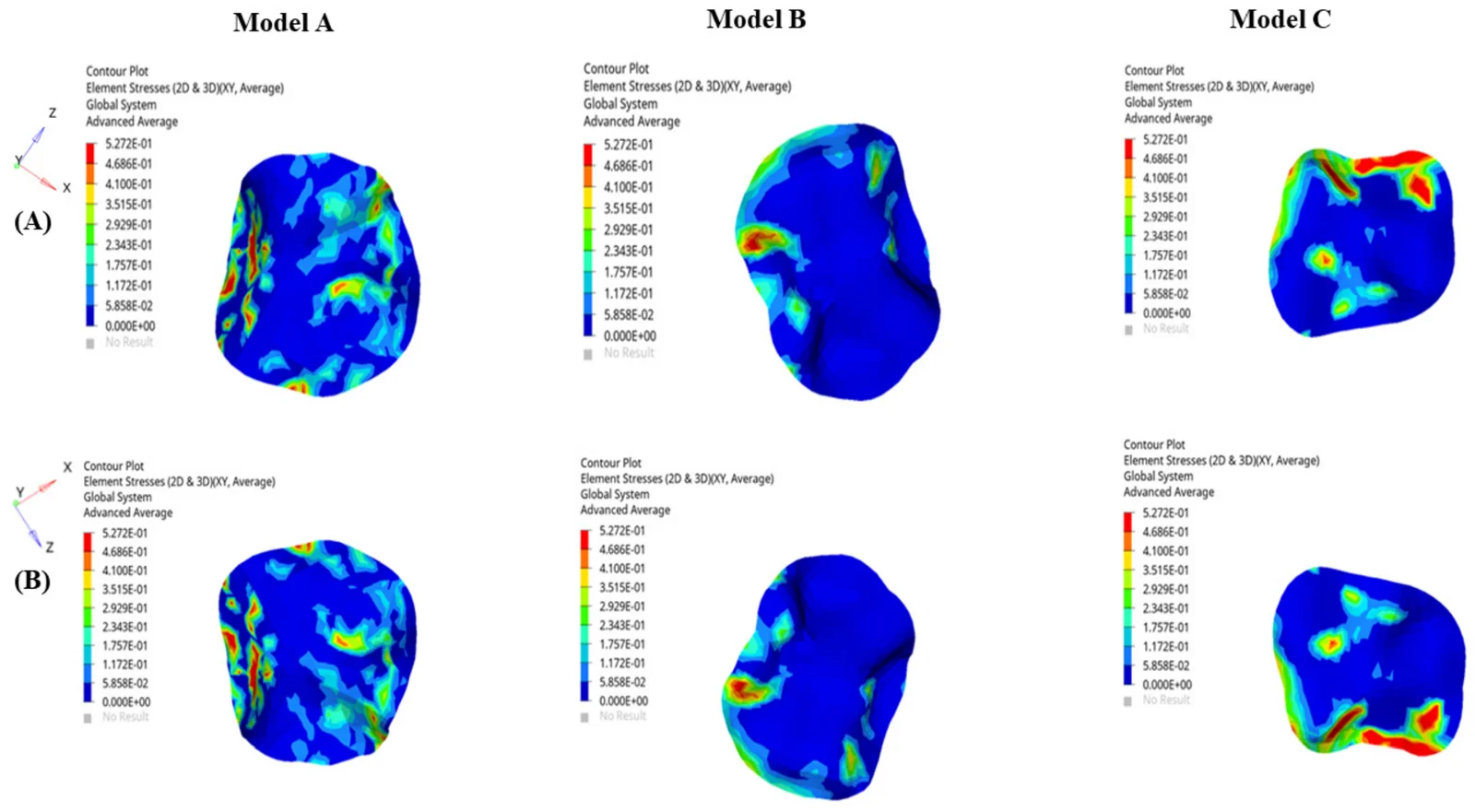
Results from FEA on models A, B, and C: shear stress (τ_xy_) distribution (MPa) at the adhesive interface layer: (**A**) lower surface; (**B**) upper surface. The colour scale was properly selected to allow comparisons among the models.

**Figure 5:**
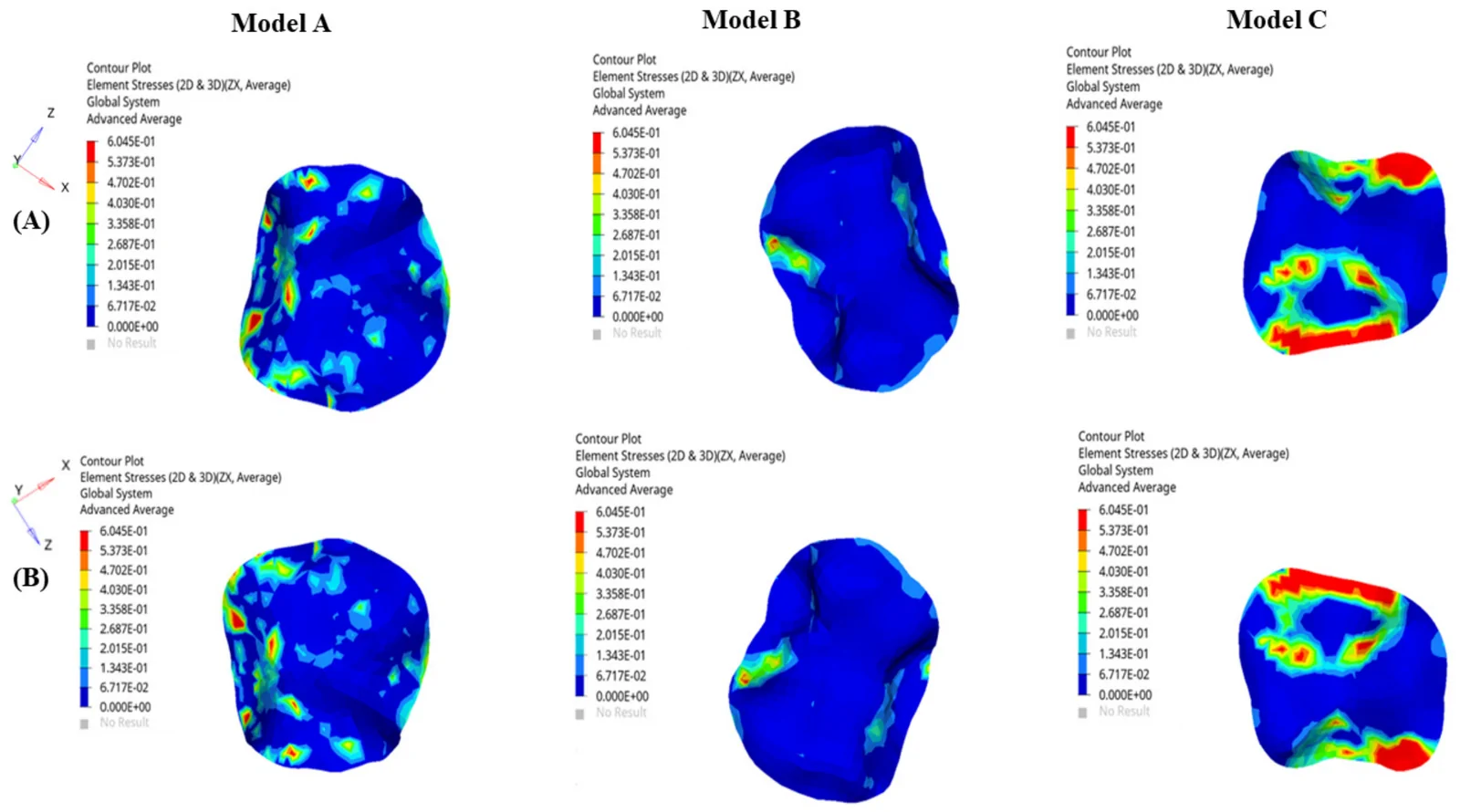
Results from FEA on models A, B, and C: shear stress (τ_zx_) distribution (MPa) at the adhesive interface layer: (**A**) lower surface; (**B**) upper surface. The colour scale was properly selected to allow comparisons among the models.

**Table 1:** Results from mechanical measurements on the different groups: tensile bond strength (F_max_) reported as mean value ± standard deviation.

Group	F_max_ (N)
A	403.7 ± 37.5
B	340.4 ± 32.7
C	324.8 ± 33.3

## Data Availability

The original contributions presented in this study are included in the article. Further inquiries can be directed to the corresponding authors.

## Abbreviations

The following abbreviations are used in this manuscript:

IPBR	Indirect Partial Bonded Restorations
FEA	Finite Element Analysis
IDS	Immediate Dentin Sealing
